# Human monocytes downregulate innate response receptors following exposure to the microbial metabolite n‐butyrate

**DOI:** 10.1002/iid3.184

**Published:** 2017-07-06

**Authors:** Felix Lasitschka, Thomas Giese, Marco Paparella, Stefan R. Kurzhals, Guido Wabnitz, Katrin Jacob, Judith Gras, Konrad A. Bode, Anne‐Kristin Heninger, Timea Sziskzai, Yvonne Samstag, Cornelia Leszinski, Bettina Jocher, Mohammed Al‐Saeedi, Stefan C. Meuer, Jutta Schröder‐Braunstein

**Affiliations:** ^1^ Institute of Pathology University Hospital Heidelberg Im Neuenheimer Feld 224 69120 Heidelberg Germany; ^2^ Institute of Immunology University Hospital Heidelberg Im Neuenheimer Feld 305 69120 Heidelberg Germany; ^3^ Department of Infectious Diseases, Medical Microbiology and Hygiene University Hospital Heidelberg Im Neuenheimer Feld 324 69120 Heidelberg Germany; ^4^ Department of Anesthesiology University Hospital Heidelberg Im Neuenheimer Feld 110 69120 Heidelberg Germany; ^5^ Department of Surgery St. Vincentius Hospital Holzstr. 4a 67346 Speyer Germany; ^6^ Department of Surgery University Hospital Heidelberg Im Neuenheimer Feld 110 69120 Heidelberg Germany

**Keywords:** Human, inflammation, monocytes/macrophages, mucosa

## Abstract

**Introduction:**

Hyporesponsiveness of human lamina propria immune cells to microbial and nutritional antigens represents one important feature of intestinal homeostasis. It is at least partially mediated by low expression of the innate response receptors CD11b, CD14, CD16 as well as the cystine‐glutamate transporter xCT on these cells. Milieu‐specific mechanisms leading to the down‐regulation of these receptors on circulating monocytes, the precursor cells of resident macrophages, are mostly unknown.

**Methods:**

Here, we addressed the question whether the short chain fatty acid n‐butyrate, a fermentation product of the mammalian gut microbiota exhibiting histone deacetylase inhibitory activity, is able to modulate expression of these receptors in human circulating monocytes.

**Results:**

Exposure to n‐butyrate resulted in the downregulation of CD11b, CD14, as well as CD16 surface expression on circulating monocytes. XCT transcript levels in circulating monocytes were also reduced following exposure to n‐butyrate. Importantly, treatment resulted in the downregulation of protein and gene expression of the transcription factor PU.1, which was shown to be at least partially required for the expression of CD16 in circulating monocytes. PU.1 expression in resident macrophages in situ was observed to be substantially lower in healthy when compared to inflamed colonic mucosa.

**Conclusions:**

In summary, the intestinal microbiota may support symbiosis with the human host organism by n‐butyrate mediated downregulation of protein and gene expression of innate response receptors as well as xCT on circulating monocytes following recruitment to the lamina propria. Downregulation of CD16 gene expression may at least partially be caused at the transcriptional level by the n‐butyrate mediated decrease in expression of the transcription factor PU.1 in circulating monocytes.

## Introduction

Intestinal homeostasis in the presence of large amounts of foreign antigens in the gut lumen is maintained by a hyporesponsive differentiation state of local immune cells 1. In particular, downregulation of innate response receptors such as CD14, CD11b, and CD16 on resident lamina propria macrophages (LPMO) when compared to peripheral blood monocytes (PBMO) 2, 3, 4 likely represents an important mechanism to secure homeostasis by preventing unwanted chronic inflammatory responses of mucosal immune cells to both, bacterial or nutritional antigens. CD14 together with Toll‐like receptor 4 (TLR4) and myeloid differentiation protein 2 (MD2) forms the lipopolysaccharide (LPS) receptor complex and hence contributes to the cellular immune response toward this bacterial compound (reviewed in Ref. 5). CD11b, a subunit of the complement receptor 3 (CR3), is essentially involved in complement‐mediated phagocytosis of microbes 6. Binding of the complement factor iC3b to CD11b is enhanced by interaction of CD11b with CD16 7, a low affinity IgG receptor, which is also crucially involved in phagocytosis 8, 9. Phagocytosis does not only represent an important step for the destruction of pathogens but also elicits an inflammatory response in myeloid cells by promoting induction of cytokine expression or inflammasome activation (reviewed in Ref. 10, 11). Furthermore, the impaired capacity of lamina propria T lymphocytes to mount an adaptive proliferative response to T cell antigen receptor stimulation as induced by antibody‐mediated crosslinking of the antigen receptor subunit CD3 when compared to peripheral blood T lymphocytes is at least partially attributed to the low expression of the cystine /glutamate transporter xCT on LPMO 12.

The origin of tissue macrophages has recently been addressed in seminal fate mapping and parabiosis studies (reviewed in Ref. 13). It was shown that murine adult macrophage populations of many organs such as the skin, lung, and brain are mostly derived from embryonic or fetal precursors, which do not originate from hematopoietic stem cells and seed those organs prior to birth 8, 14, 15, 16, 17. Furthermore, there is evidence that resident macrophages in those tissues are long‐lived and maintained by self‐renewal 18 with only minor contributions from circulating monocytes 19. In contrast to most other tissues, however, the intestinal lamina propria has been shown to harbor resident macrophages that originate from embryonic precursors during the neonate period, but are replaced during the first weeks after birth and subsequently maintained by circulating monocytes under steady‐state conditions 20. Notably, distinct populations of resident intestinal macrophages reflecting successive stages of the differentiation process from monocytes as observed in mice could also be observed in humans 21. This suggests that circulating monocytes contribute to the resident lamina propria macrophage pool also in humans. In support of this notion, mucosal cells constitutively produce mediators known to attract monocytes such as TGF‐β 4 and CXCL12 22.

So far, micromilieu‐specific factors driving the differentiation of human monocytes into lamina propria macrophages including the downregulation of innate response receptors and xCT on PBMO remain largely unknown. It has been reported that intestinal stromal cell derived products are able to downregulate CD14 and CD16 on human peripheral blood monocytes 4. Furthermore, TGF‐b has been shown to downregulate LPS‐induced cytokine production in human monocytes 4 and to contributes to the differentiation process of murine intestinal macrophages 23.

Here, we addressed the question whether the short chain fatty acid n‐butyrate, a fermentation product of the mammalian gut microbiota with histone deacetylase (HDAC) inhibitory activity, is able to modulate expression of these receptors in primary human PBMO. N‐butyrate is detectable in the gut lumen in high concentrations (11–24 mM) 24, 25. While serving as the main energy source for intestinal epithelial cells 26, 27, it also appears to be able to access the underlying mucosal tissue as well as the blood circulation since it is detectable in portal vein blood at higher concentrations than in peripheral blood 28. Importantly, n‐butyrate has recently been implicated in the modulation of the intestinal immune cell compartment 29, 30, 31 through induction of regulatory T cells.

To explore the effect of n‐butyrate on expression levels of CD14, CD11b, and CD16 as well as xCT, PBMO were exposed to this short chain fatty acid. Subsequently, surface and/or gene expression of the latter receptors was analyzed using flow cytometry and quantitative polymerase chain reaction (qPCR), respectively.

Given that 1 the promoter/enhancer regions of the genes encoding most of these receptors contain consensus sequences for the transcription factor PU.1 and 2 constitutive PU.1 expression has been shown to be downregulated by HDAC inhibitors in murine myeloid cell lines 32, 33, we further examined whether n‐butyrate may affect expression of these surface receptors in primary human PBMO through regulation of PU.1. To this end, we analyzed the modulation of PU.1 expression by n‐butyrate in the latter cell population. Secondly, we determined whether expression of these surface receptors in PBMO depends on PU.1 activity. Finally, the expression level of PU.1 was analyzed in situ in lamina propria macrophages of both, normal and inflamed colonic mucosa.

## Materials and Methods

### Patient details

Peripheral blood was collected from healthy individuals (age ranging from 27 to 62 years; 64.3% female, 35.3% male donors) by peripheral venous puncture. Individuals were excluded from the study if they 1 had a medical history of chronic or other severe illnesses, 2 had undergone major surgery within the last 4 months before blood withdrawal, 3 had suffered from febrile diseases or diarrhea during the last 4 weeks before venipuncture, 4 had undergone minor surgical procedures (e.g., dental surgery) within 1 week of venipuncture, 5 reported to acutely have symptoms of common cold or other health problems at the time point of venipuncture or 6—in case of females—reported to be pregnant. For in situ analysis, tissues were derived from patients undergoing resection for localized colon cancer, functional colonic disorder, mesenterial tumor or benign colonic disease, and were taken from histological normal, non‐involved areas of such specimens. Furthermore, inflamed tissue was obtained from patients undergoing bowel resection due to ulcerative colitis (UC). Except for the medical conditions described above, patients participating in this study did not suffer from any other co‐morbidities. Fresh tissue samples were immediately frozen in liquid nitrogen. All studies are approved by the ethical committee of the University of Heidelberg and conducted according to the principles expressed in the Helsinki Declaration (Ethical vote No. 024/2003).

### Antibodies

For flow cytometric analysis the following monoclonal antibodies (Ab) were obtained from BD Biosciences (Heidelberg, Germany): CD3/CD19/CD20 APC‐H7, CD11b PE‐CF594, CD14 V450, CD16 APC, CD33 BV711, CD45 AF700, CD56 PE‐Cy5, HLA‐DR V500. For InFlow microscopic analysis, PE‐labeled CD3 mouse mAb (IgG_1_; BD Biosciences), unconjugated CD33 mouse mAb (IgG_1_; BD Biosciences) as well as PU.1 rabbit pAb (IgG; Santa Cruz Biotechnology, Dallas, TX) was used. Western blot analysis was performed using the following primary antibodies: PU.1 rabbit pAb (Santa Cruz Biotechnology), STAT3 rabbit pAb (Santa Cruz Biotechnology), histone H4 mouse mAb (IgG_2a_, Merck Millipore, Darmstadt, Germany), acetyl‐histone H4 rabbit pAb (Millipore). For immunofluorescence analysis tissue sections were stained with CD68 mouse mAb (IgG_1_; BD) and PU.1 rabbit pAb (IgG; Santa Cruz Biotechnology).

### Cell isolation from peripheral blood

Peripheral blood mononuclear cells (PBMC) were isolated by Ficoll density gradient centrifugation. Peripheral blood monocytes (PBMO) were enriched by plastic adherence for 2 h. Alternatively, PBMO were purified by negative selection using magnetic beads (MACS, Miltenyi Biotec GmbH, Bergisch‐Gladbach, Germany).

### N‐butyrate treatment of PBMC/PBMO

PBMC or PBMO (enriched by plastic adherence) were cultured in RPMI 1640 (Thermo Fisher Scientific, Waltham, MA)/10%FCS (Sigma, St.‐Louis, MO)/2% glutamine (Thermo Fisher Scientific)/antibiotics (Thermo Fisher Scientific) in the presence or absence of n‐butyrate (0.5 mM, 1 mM; Sigma) for 4 h and/or 24 h. Subsequently, cells were lysed and subject to Western blotting or gene expression analysis. Alternatively, they were further processed for flow cytometric or InFlow microscopic analysis (see section Flow Cytometry and section InFlow Microscopy). Where indicated, the culture was performed in the presence of the pan‐caspase inhibitor zVAD‐fmk (Merck Millipore).

### Flow cytometry

1 × 10^5^ to 1 × 10^6^ PBMC were incubated with a combination of up to 11 different fluorochrome‐labelled antibodies. PBMO were identified as CD45^+^ lineage (lin)^−^ (CD3/CD19/CD20/CD56^−^) CD33^+^ HLA‐DR^+^ cells. Flow cytometric analysis was performed using an LSR II (BD Biosciences) and FACSDiva v6 software (BD Biosciences). Doublets were identified by plotting Forward Scatter—Area (FSC‐A) versus Forward Scatter—Height (FSC‐H) and excluded from the analysis. As a negative control the (auto)fluorescence of the myeloid cell populations (CD45^+^ CD3/CD19/CD20/CD56^−^ CD33^+^) was determined.

Where indicated annexin V staining with/without propidium iodide staining was performed using the FITC Annexin V Apoptosis Detection Kit I (BD Biosciences). The procedure was conducted according to the manufacturer's instructions.

### Gene expression analysis

Cells were collected in 400 μl lysis buffer from the MagnaPure mRNA Isolation Kit I (Roche Diagnostics, Mannheim Germany) and messenger ribonucleic acid (mRNA) was isolated with the MagnaPure‐LC device using the mRNA‐I standard protocol. Tissue samples were disrupted with the aid of a RiboLyser device (ThermoHYBAID, Heidelberg, Germany) in lysing matrix “D” tubes (Q‐BIOgen, Heidelberg, Germany) containing 400 μl lysis buffer from the MagnaPure mRNA Isolation Kit II (Roche Diagnostics). A total of 300 μl of the lysate was collected and mixed with 600 μl capture buffer containing oligo‐dT. After centrifugation at 13000 rpm for 5 min, 880 μl of this mix was transferred into a MagnaPure sample cartridge and mRNA was isolated with the MagnaPure‐LC device using the mRNA‐II standard protocol. mRNA was reverse transcribed using AMV‐RT and oligo‐ (dT) as primer (First Strand cDNA Synthesis Kit, Roche) according to the manufactures protocol. Primer sets optimized for the LightCycler (RAS, Mannheim, Germany) were developed and provided by SEARCH‐LC GmbH (Heidelberg, Germany). The PCR was performed with the LightCycler FastStart DNA Syber Green I kit (RAS) according to the protocol provided in the parameter specific kits. To correct for differences in the content of mRNA, the calculated transcript numbers were normalized according to the expression of the housekeeping gene peptidylprolyl isomerase B (*PPIB*). Values were thus given as transcripts per 1000 transcripts of *PPIB*.

### Preparation of cell extracts and Western blotting

Whole cell extracts of PBMO were prepared and analyzed by immunoblotting as previously described 34, 35. Densitometric analysis was performed using a scanner (GS‐800; Bio‐Rad, Munich, Germany) and Quantity One Software (Bio‐Rad). Expression levels of PU.1 and STAT3 were normalized based on histone H4 levels. Levels of both transcription factors in untreated PBMO were set to 100%. Expression levels of all other conditions were calculated as % of expression levels relative to untreated PBMO.

### Immunoenzyme and double immunofluorescence staining

Immunoenzyme stainings of PU.1 (rabbit anti‐PU.1 IgG; Santa Cruz Biotechnology) were performed on cryostat sections (4 µm) of fresh frozen tissues, which were post‐fixed in 2% paraformaldehyde and further processed by use of the paraformaldehyde‐saponin‐procedure in combination with the standard alkaline phosphatase anti‐alkaline phosphatase technique (Dako, Glostrup, Denmark) according to a previously published protocol 36. The primary Ab was added overnight at room temperature. A mouse anti‐rabbit mAb, 1/50 (Dako), was used as a secondary reagent (30 min at room temperature). Naphthol AS‐biphosphate (Sigma) with New‐fuchsin (Merck, Darmstadt, Germany) was used as the substrate for alkaline phosphatase 37.

Double immunofluorescence staining was performed on freshly cut cryostat sections from normal gut and ulcerative colitis. Sections were fixed in acetone and methanol, before being permeabilized in PBS/0.05% bovine serum albumin (Aurion) containing 0.5% Saponin (Sigma) and labelled with un­conjugated mouse anti‐CD68 mAb (IgG_1_; BD Biosciences) and unconjugated rabbit anti‐PU.1 (IgG; Santa Cruz Technology) followed by incubation with a Cy3‐conjugated goat anti‐mouse ab (1/250; Dianova, Hamburg, Germany) and a Cy5‐conju­gated donkey anti‐rabbit ab (1/250; Dianova). For negative controls isotype‐ and concentration‐matched unconjugated mouse IgG_1_ (1/100; Dianova) and rabbit IgG (1/1000; Dianova) were used. Slides were viewed with a Laserscan microscope using suitable filter combinations provided by the manufacturer (Leica Microsystems, Mannheim, Germany).

### PU.1 knock‐down

A small interfering RNA (siRNA) against human PU.1 (ON‐TARGETplus siRNA; J‐010537‐06) and nonsilencing siRNA (ON‐TARGETplus Non‐targeting pool D‐001810‐10‐05) were purchased from Thermo Scientific (Waltham, MA). PBMO (purified by negative selection using MACS) were transfected using the HVJ Envelope Vector Kit GenomONE‐Neo ex (Cosmo Bio, Tokyo, Japan) according to the manufacturer's instructions. Briefly, HVJ‐E (0.25 AU) was mixed with 10 μl of siRNA solution (30 μM) and 2 μl of Reagent B. After centrifugation, the precipitate was resuspended in a mixture of 30 μl buffer and 5 μl Reagent C. An aliquot (35 μl) of this siRNA‐HVJ‐E mixture was added to 2 × 10^6^ PBMO and centrifuged at 10,000 *g* for 20 min at 4°C. The supernatant was removed, and the cells were resuspended in 2 ml of culture medium. After 24 h of culturing in RPMI 1640 (Thermo Fisher Scientific)/10%FCS (Sigma)/2% glutamine (Thermo Fisher Scientific)/antibiotics (Thermo Fisher Scientific), the cells were harvested and used for gene and protein expression analysis as described above.

### InFlow microscopy

For fluorescence staining, PBMC were fixed in ice‐cold Cytofix/Cytoperm solution (BD Biosciences), washed in cold PBS/0.5% bovine serum albumin (Aurion, Wageningen, NL) containing 0.5% Saponin (Sigma) and labelled with Hoechst 33342 (1/10000; Thermo Fisher Scientific), Annexin V FITC, PE‐conjugated mouse anti‐CD3 mAb (IgG_1_; BD Biosciences), unconjugated mouse anti‐CD33 mAb (IgG_1_; BD Biosciences) as well as unconjugated rabbit anti‐PU.1 (IgG; Santa Cruz Biotechnology). Binding of unlabeled antibodies was detected using a biotinylated goat anti‐mouse IgG_1_ mAb (1/250; Dianova) in combination with PE‐TexasRed‐conjugated streptavidin (1/100;Thermo Fisher Scientific) and a Cy5‐conjugated donkey anti‐rabbit IgG (1/250; Dianova) as secondary antibodies respectively. PBMO were identified as Annexin^−^ CD33^+^ CD3^−^ cells.

Image files were automatically acquired in flow with an ImageStream imaging cytometer (Amnis, Seattle, WA). Single color controls were used to calculate the spectral crosstalk matrix. Compensated image files were analyzed with IDEAS 3.0 (Amnis). The expression of PU.1 or CD33, respectively, was calculated by the addition of the intensity values of all pixels in the respective image.

### Statistical analysis

Where indicated, data are presented as the mean ± standard error of the mean (SEM). Statistical analysis was performed using the non‐parametric Friedman test in combination with Dunn's multiple comparison test (Prism V, GraphPad Software, Inc., San Diego).

## Results

### The bacterial metabolite n‐butyrate downregulates expression of innate response receptors as well as the cystine‐glutamate transporter xCT in primary human PBMO

**Figure 1:**
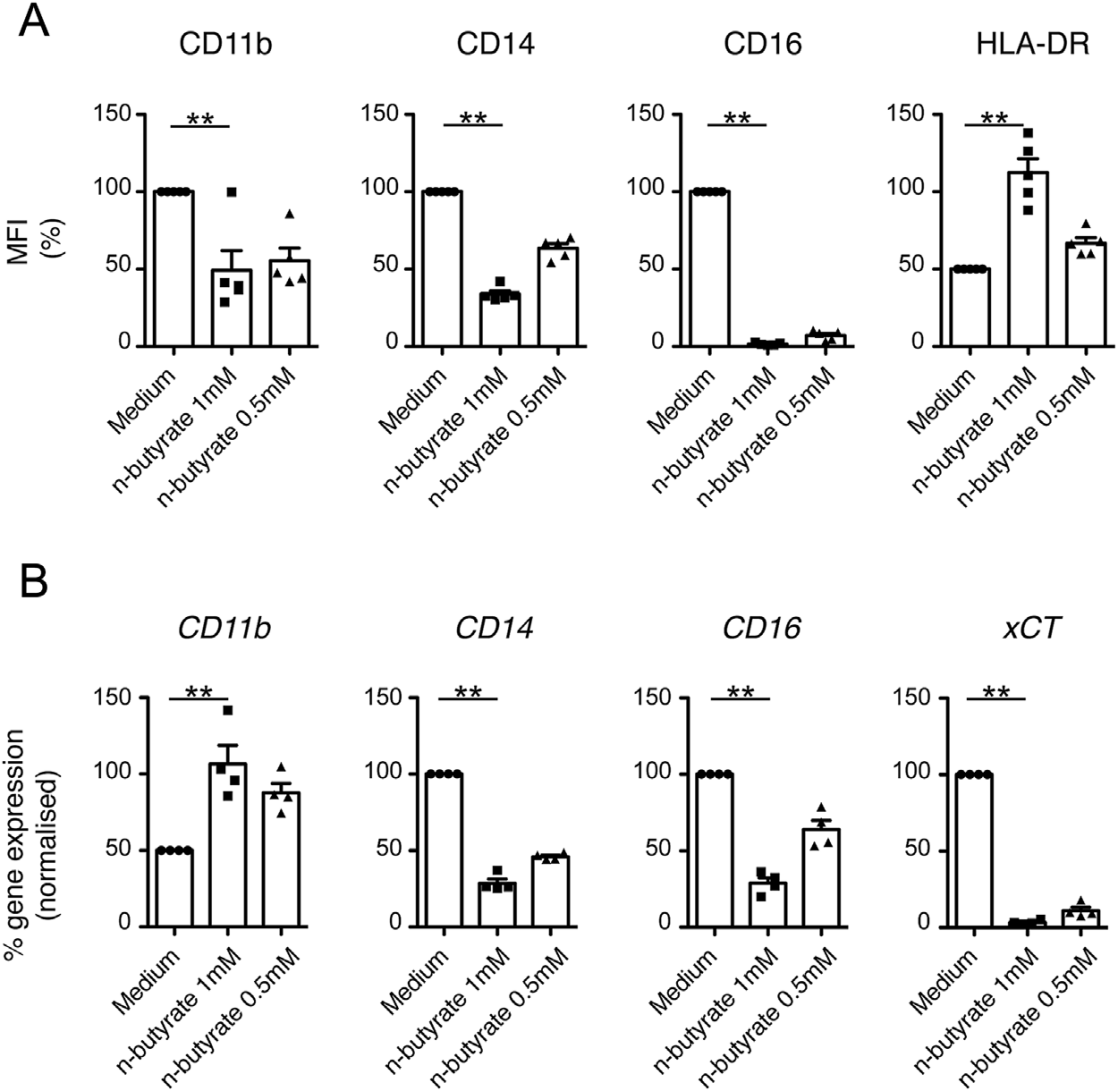
N‐butyrate downregulates expression of innate response receptors as well as the cysteine‐glutamate transporter xCT in primary human PBMO. PBMC were cultured in the absence or presence of different concentrations of n‐butyrate (1 mM, 0.5 mM). (A) After 24 h of culture, PBMC were harvested, stained with the appropriate antibodies, and subject to flow cytometric analysis. A gate was set on CD45^+^ lineage^−^ (CD3/CD19/CD20/CD56^−^) CD33^+^ HLA‐DR^+^ annexin V^−^ PBMO in order to determine surface expression levels of CD11b, CD14, CD16 as well as HLA‐DR specifically on this cell population. Results are presented as % mean fluorescence intensity (MFI) of untreated control (medium). Shown is the mean ± SEM as well as individual data points of 5 independent experiments. (B) After 4 h of culture, expression of the genes encoding *CD11b*, *CD14*, *CD16*, and *xCT* was determined by qPCR. Normalized transcript levels in n‐butyrate treated PBMO were presented as % expression of untreated control (medium). Shown is the mean ± SEM as well as individual data points of 3–4 independent experiments.

In comparison to PBMO, LPMO express low levels of the innate response receptors CD11b, CD14, and CD16 2, 3, 4. Accordingly, low expression of these receptors has been observed in normal mucosa in situ when compared to inflamed mucosa of patients suffering from inflammatory bowel disease 38, 39, 40, 41, 42. In order to determine a potential regulation of the expression of these receptors by the bacterial metabolite n‐butyrate in PBMO, peripheral blood mononuclear cells (PBMC) were exposed to this compound at concentrations of 1 and 0.5 mM, respectively, for 24 h. Subsequent flow cytometric analysis of PBMO (identified as CD45^+^ lineage^−^ CD33^+^ HLA‐DR^+^ cells within the PBMC population) revealed a dose‐dependent downregulation of CD11b, CD14, and CD16 in the presence of n‐butyrate, while the expression of HLA‐DR, a receptor highly expressed on LPMO 43, 44, was upregulated (Fig. 1A). Note that the analysis was confined to annexin V‐negative PBMO in order to avoid potential confounding effects due to n‐butyrate‐induced apoptosis or apoptosis due to prolonged culture 45. The number of annexin V‐positive PBMO was on average 12.4 ± 7.4% (1 mM n‐butyrate) and 13.6 ± 5.6% (0.5 mM n‐butyrate), respectively, higher following n‐butyrate treatment when compared to medium control (Fig. S1). Importantly, a similar reduction of surface expression levels of CD11b, CD14 as well as CD16 was observed when PBMO enriched by plastic adherence or purified by negative magnetic selection were exposed to n‐butyrate (Fig. S2A and B). This suggests that butyrate mediates the downregulation of these receptors by directly affecting monocytes.

Given its HDAC inhibitory activity, n‐butyrate is capable of modulating gene expression for example, through acetylation‐induced alteration of the chromatin structure or transcription factor activity 46, 47. In order to determine whether downregulation of surface expression of CD11b, CD14, and CD16 on PBMO by n‐butyrate is associated with decreased expression levels of the corresponding genes, transcript levels of these genes were determined in untreated vs. n‐butyrate treated PBMO by qPCR. Furthermore, the effect of n‐butyrate on mRNA levels of an additional surface receptor known to be downregulated in LPMO and involved in the regulation of the adaptive immune response, the cystine‐glutamate transporter xCT 12, was analyzed. As shown in Figure 1B, mRNA levels of *CD14*, *CD16*, and *xCT* were decreased while that of *CD11b* were increased in PBMO after 4 h (Fig. 1B) of n‐butyrate exposure when compared to the untreated control.

### The bacterial metabolite n‐butyrate downregulates the expression of the transcription factor PU.1 in human PBMO

**Figure 2:**
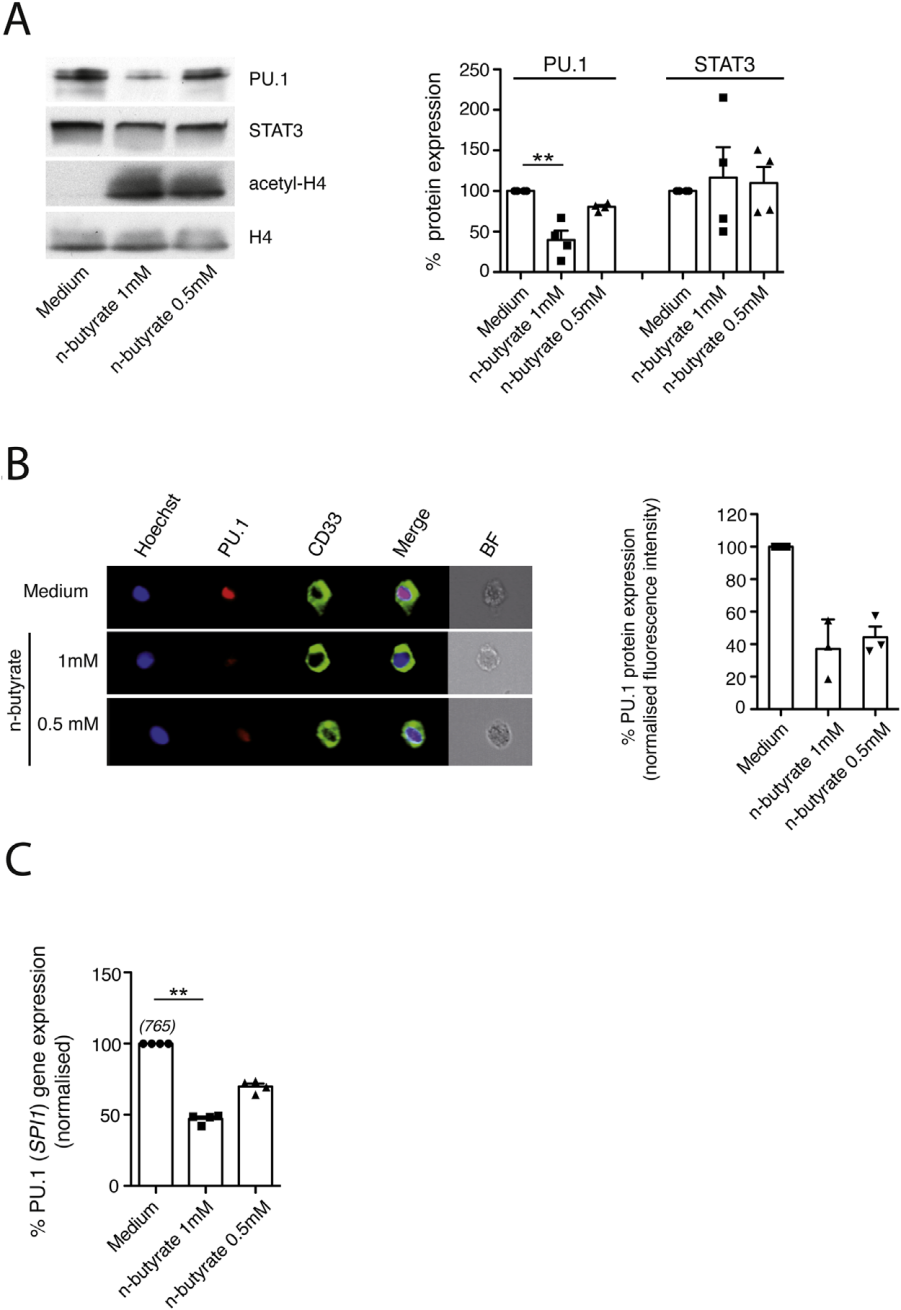
Exposure to n‐butyrate results in downregulation of PU.1 expression in primary human PBMO. PBMO were cultured in the absence or presence of different concentrations of n‐butyrate (1 mM, 0.5 mM). (A) After 24 h of culture in the presence of the pan‐caspase inhibitor z‐VAD‐fmk, protein expression of PU.1, STAT3 and histone H4 (loading control) as well as the acetylation state of histone H4 (acetyl‐H4) was determined in PBMO lysates by Western blotting using PU.1, STAT3, histone H4, and acetylated histone H4 specific antibodies (left panel). Corresponding densitometric quantification of protein expression levels was performed as described in Materials and Methods (right panel). Shown are the means ± SEM as well as individual data points of 5 independent experiments. (B) Cells were stained for Hoechst 33342, CD33, annexin V, and PU.1 and analyzed by InFlow microscopy. At least 10,000 images were collected and gating was performed to generate a set of single, in‐focus cell images. A region was created on CD33^+^ annexin V^−^ PBMO. Left panel: Representative images of PU.1 in annexin V^−^ PBMO (−/+ n‐butyrate) were selected and are shown with the overlay images of PU.1, Hoechst 33342 and CD33 (termed merge) in the 4th column. BF: bright field. Right panel: PU.1 expression (measured as fluorescence intensity) in annexin V^−^ untreated PBMO (Medium) was set to 100%. PU.1 expression levels in n‐butyrate (1 mM, 0.5 mM) treated PBMO was calculated as % expression of untreated control. Shown are the means ± SEM as well as individual data points of 3 independent experiments. (C) After 4 h of culture, expression of the gene encoding PU.1 (SPI1) was determined by qPCR. Normalized transcript levels in n‐butyrate (1 mM, 0.5 mM) treated PBMO were presented as % expression of untreated control (medium). Shown are the means ± SEM as well as individual data points of 3–4 independent experiments. Numbers in brackets indicate the mean transcript numbers (normalized to PPIB) of 3–4 independent experiments.

The promoter/enhancer region of the gene encoding human CD11b has been shown to contain consensus sequences for the transcription factor PU.1, which is constitutively expressed in myeloid cells 48, 49. In addition, CD14 gene expression is regulated by the transcription factor KLF4, which has also been demonstrated to be a target of PU.1 50. Importantly, expression of PU.1 was shown to be downregulated by histone HDAC inhibitors including n‐butyrate in murine macrophage cell lines 32, 33 and murine bone marrow stem cells 51. In order to determine whether n‐butyrate is able to modulate the constitutive PU.1 expression in primary human PBMO, the latter cell population was exposed for 24 h to this short chain fatty acid. The culture was performed in the presence of zVAD‐fmk, a pan‐caspase inhibitor, which is known to counteract apoptosis 52, 53. As determined by immunoblotting of cell lysates, n‐butyrate treatment (0.5 mM, 1 mM) resulted in a reduction of PU.1 expression in PBMO, while the expression of the transcription factor STAT3 was not significantly affected indicating the specificity of the n‐butyrate mediated effect (Fig. 2A). Furthermore, n‐butyrate caused an upregulation of histone H4 acetylation in PBMO, which is in accordance with its HDAC inhibitory activity. N‐butyrate induced downregulation of PU.1 protein expression could be confirmed in annexin V‐negative PBMO on the single cell level by inFlow microscopy (Fig. 2B). Note that complete co‐localization of the PU.1 signal with the nucleic acid stain Hoechst33342 in PBMO under all experimental conditions indicates a constitutive nuclear localization of this transcription factor in these cells, which is in agreement with previous results 54.

Compatible with a regulation of PU.1 expression on the level of gene expression, *PU.1* (gene symbol *SPI1*) mRNA levels were reduced in n‐butyrate treated versus untreated PBMO after 4 h of culture (Fig. 2C).

### CD16 gene expression in human PBMO is regulated by the transcription factor PU.1

**Figure 3:**
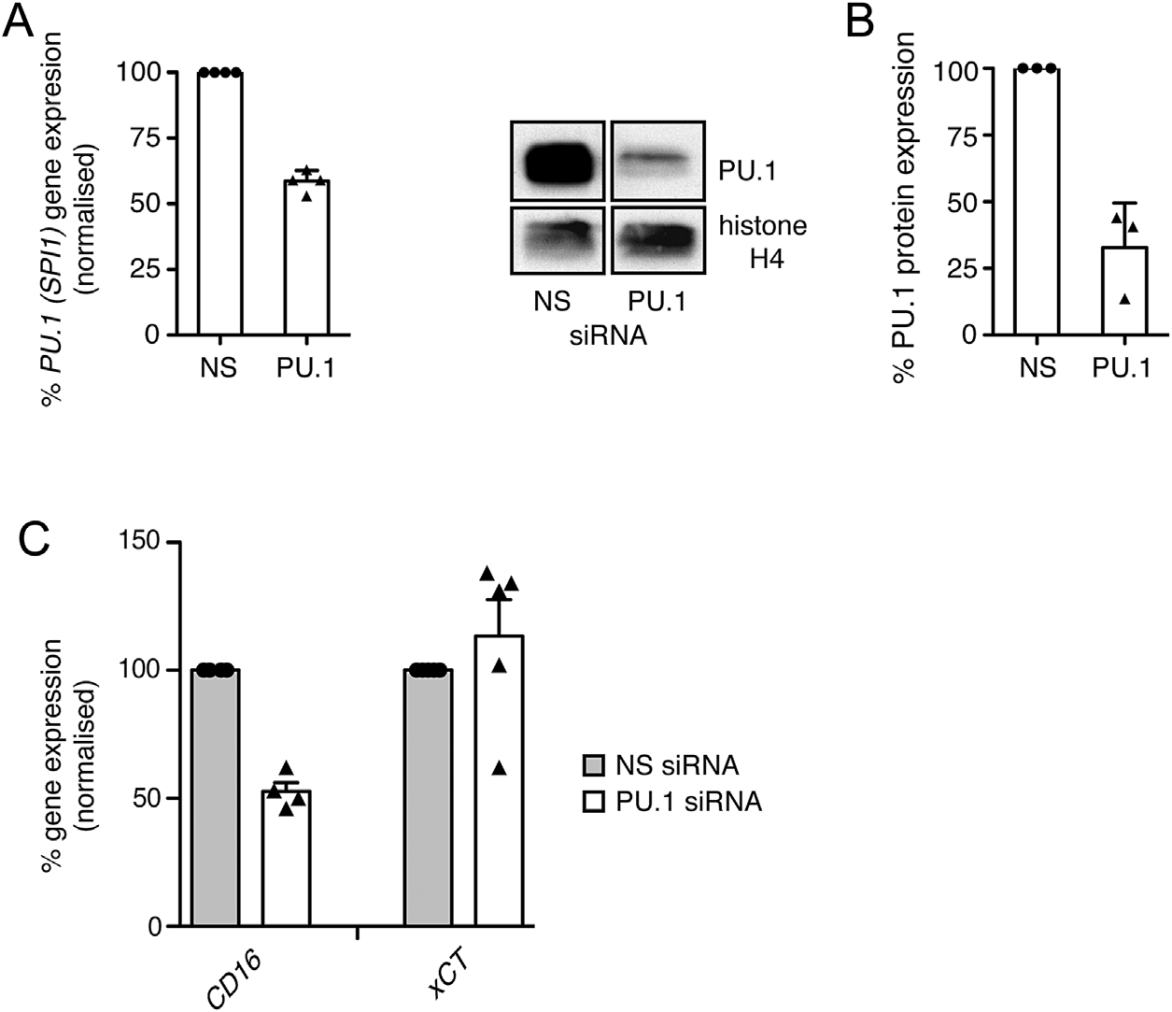
PU.1 knock‐down results in reduced CD16 receptor gene expression in PBMO. PBMO were transfected with PU.1‐specific (PU.1) or non‐silencing (NS) siRNA. (A) After 24 h, PU.1 gene expression levels were analyzed by qPCR. (B) After 24 h, protein expression of PU.1 and histone H4 (loading control) was determined in PBMO lysates by Western blotting using PU.1 and histone H4 specific antibodies (left panel). Corresponding densitometric quantification of PU.1 protein expression levels was performed as described in Materials and Methods (right panel). Shown are the means ± SEM as well as individual data points of 3–4 independent experiments. (C) Transcript levels of CD11b, CD14, CD16, and xCT in siRNA‐transfected PBMO were analyzed by qPCR and expressed as percentage based on the level in nonsilencing (NS) siRNA‐transfected cells. Shown are mean transcript numbers ± SEM as well as individual data points of 4 independent experiments.

The participation of PU.1 in the transcriptional regulation of *CD11b* and *CD14* in myeloid cells has been shown for cell lines and murine models 48, 50, 55. To explore the contribution of PU.1 to the expression of these genes as well as *CD16* and *xCT* in primary human PBMO, siRNA‐mediated PU.1 gene knock‐down was performed in the latter cell population, and its effect on transcript levels of these receptor genes was determined using qPCR. As shown in Figure 3A and B, transfection of PBMO with *PU.1* specific siRNA resulted in an average reduction of *PU.1* mRNA levels by 47% and of PU.1 protein expression by 67% when compared to treatment with non‐silencing siRNA after 24 h. Knock‐down of PU.1 caused a reduction of *CD16* mRNA levels (47%) indicating that in this cell population expression of this receptor gene is at least partially dependent on PU.1 (Fig. 3C). In contrast, *xCT* as well as *CD11b* and *CD14* transcript levels were not affected by PU.1 knock‐down under the experimental conditions employed (Fig. 3C and data not shown).

### The transcription factor PU.1 is expressed at low levels in human LPMO in situ under homeostatic conditions

**Figure 4:**
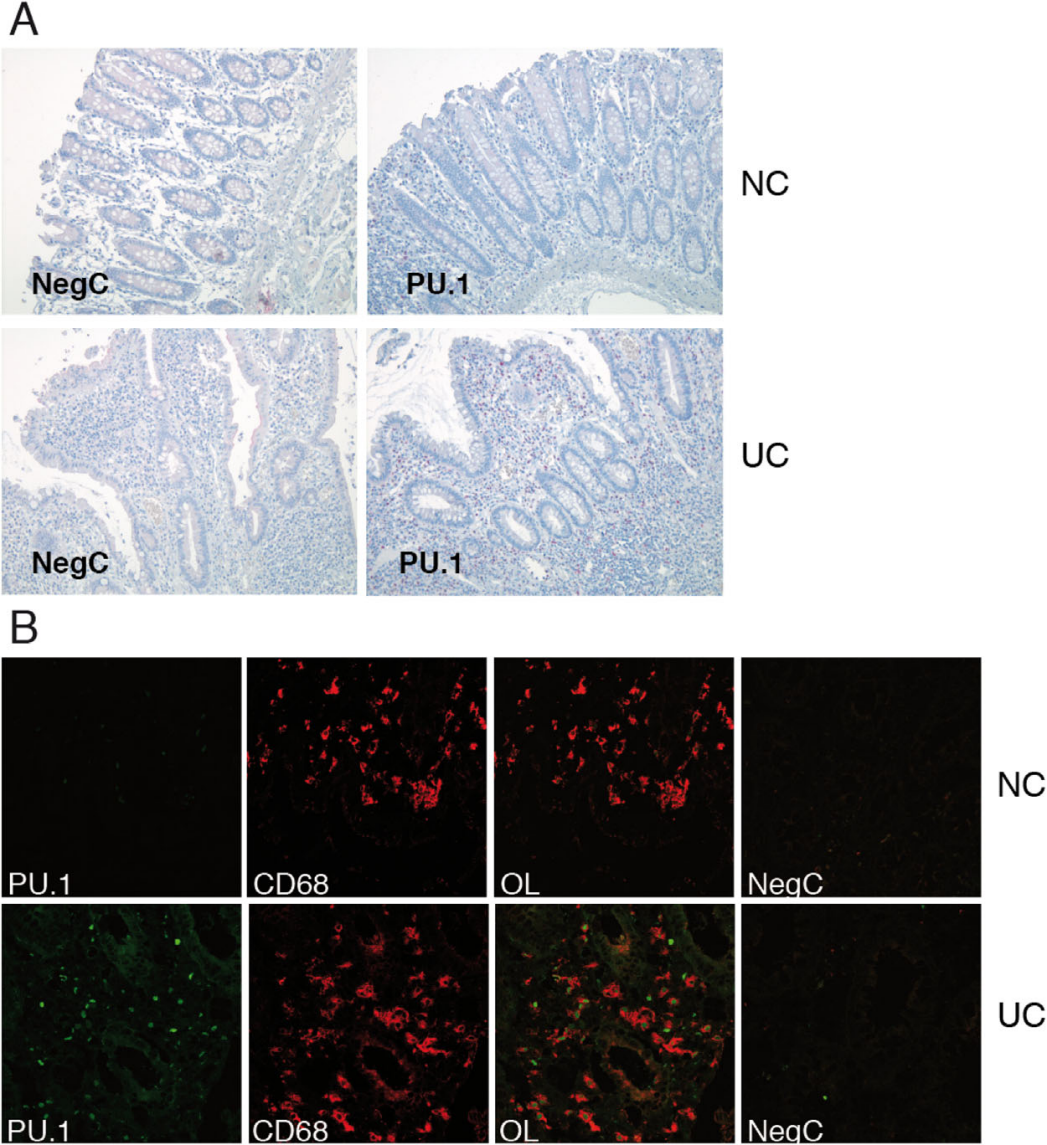
In situ expression of the transcription factor PU.1 in normal and inflamed colon mucosa. (A) Tissue sections of normal (NC) and inflamed colonic mucosa (UC) were subject to immunostaining for PU.1 (red staining). The result of 1 representative experiment out of 3 is shown. Negative control (NegC): rabbit IgG was employed as first antibody. Paraformaldehyde‐saponin procedure, alkaline phosphatase; magnification, ×30. (B) Immunofluorescence staining of PU.1 (green), and CD68 (red) in normal colonic mucosa (NC) and a case of inflamed colon in ulcerative colitis (UC). The result of 1 representative experiment out of 3 is shown. OL, overlay; magnification, ×40.

Given the n‐butyrate mediated downregulation of PU.1 expression in PBMO, we addressed the physiological relevance of this finding by analysing PU.1 protein levels in LPMO in situ employing immunohistology. Figure 4A shows that immune reactivity of lamina propria mononuclear cells for PU.1 in normal (healthy) gut mucosa (NC) was weak. In contrast, in specimens obtained from patients suffering from ulcerative colitis (UC) in situ expression of PU.1 by mononuclear cells within the inflamed lamina propria was high. Immunofluorescence stainings confirmed the enhanced PU.1 expression by CD68^+^ lamina propria macrophages in UC versus normal intestinal mucosa (Fig. 4B).

## Discussion

The commensal microbiota of the intestine has been implicated in the regulation of diverse functions of the host organism such as metabolism and immune defense 56, 57, 58, 59. The molecular mechanisms underlying its communication with host cells are only partially known.

One metabolite of intestinal commensal micro‐organisms 60, the short chain fatty acid n‐butyrate, has recently been implicated in the modulation of the intestinal immune cell compartment. It has been demonstrated to promote the generation of regulatory T cells in the intestinal mucosa of mice 29, 31 thereby supporting the maintenance of local immune homeostasis. Furthermore, n‐butyrate was shown to downregulate LPS‐induced cytokine and chemokine expression in murine bone marrow derived and lamina propria macrophages 30. On the molecular level, these effects have been attributed to the ability of n‐butyrate to inhibit histone deacetylases (HDAC) 61. In line with these findings in murine models, Lührs et al. 62 reported that n‐butyrate treatment of patients suffering from ulcerative colitis resulted in reduced NF‐κB activation in lamina propria macrophages, which correlated with amelioration of intestinal inflammation.

Here, we addressed the question whether n‐butyrate contributes to another important feature of intestinal immune homeostasis, namely the low expression of innate immune response receptors (CD11b, CD14, CD16) as well as xCT, the light chain of the cystine/glutamate transporter, as observed on human LPMO 2, 4, 12. While low expression of innate response receptors impairs recognition of microbial products and hence the initiation of an innate immune response, low xCT expression on LPMO restricts the up‐take of cystine and the production of cysteine by this cell type and thereby contributes to the reduced (cysteine‐dependent) proliferative response of lamina propria T lymphocytes to antigen receptor stimulation 12, 63, 64.

We demonstrate that n‐butyrate is able to downregulate surface expression of the innate response receptors CD14, CD16, and CD11b in human PBMO. Low surface expression of CD14 and CD16 correlates with low transcript levels of the corresponding genes in the presence of n‐butyrate indicating that the former is mainly mediated by (post)transcriptional mechanisms. In contrast, (post)translational mechanisms may be responsible for the n‐butyrate induced downregulation of CD11b surface expression, which is associated with increased mRNA levels of the corresponding gene. In addition to these surface receptors, transcript levels of the cystine‐glutamate receptor xCT were observed to be downregulated in PBMO following n‐butyrate exposure. Notably, an increase in CD14^+^, CD16^+^, CD11b^+^ 65, and xCT^+^ lamina propria myeloid cells 12 correlates with a decrease in n‐butyrate producing bacteria 66 as well as diminished mucosal n‐butyrate uptake and oxidation in inflammatory bowel disease when compared to healthy conditions 67.

Acting as an HDAC inhibitor, n‐butyrate may affect gene expression by enhancing acetylation of histones and other nuclear as well as cytoplasmic or mitochondrial proteins 68. An increase in histone acetylation has mostly been associated with enhanced gene transcription due to chromatin decondensation facilitating binding of transcription factors and co‐activators 69. However, HDAC inhibitors have also been observed to repress gene expression, as it is the case in this study 30, 70. The molecular mechanisms underlying this repressive activity are less well understood: it has been demonstrated that the recruitment of the RNA polymerase II to core promoter regions of suppressed genes is reduced in the presence of HDAC inhibitors 30, 33. Furthermore, transcriptional repression of oncogenes by HDAC inhibitors has been shown to be caused by inhibition of RNA polymerase II mediated transcription elongation 71. Importantly, the binding of transcription factors to gene regulatory elements can be inhibited 70 while that of transcriptional suppressor complexes can be enhanced in the presence of HDAC inhibitors 72.

Here, we demonstrate for the first time that the transcription factor PU.1 is downregulated by n‐butyrate treatment in primary human PBMO. Furthermore, results from PU.1 knock‐down experiments indicate that expression of the surface receptor gene CD16 in this cell population correlates with PU.1 activity. Taken together, these results suggest that downregulation of PU.1 expression may contribute to the n‐butyrate‐mediated reduction of CD16 transcript levels in PBMO.

Importantly, in support of these in vitro findings, PU.1 was found to be constitutively expressed at low levels in LPMO in healthy mucosa in situ when compared to inflamed mucosa of patients suffering from ulcerative colitis.

PU.1, a member of the E twenty‐six (ETS) family of transcription factors, is exclusively expressed in cells of the hematopoietic lineage 73, 74. In mature cells, PU.1 expression is restricted to myeloid and B cells while being silenced in T lymphocytes 73. Functionally, it represents a key factor for the development of both the myeloid and lymphoid lineages as demonstrated by gene targeting studies 75, 76, 77. Thus, PU.1 deficient mice lack mature mono­cytes and macrophages due to a block in terminal myeloid differentiation 73. In humans, decreased PU.1 expression/transcriptional activity has been implicated in the pathogenesis of acute myeloid leuke­mia 73. Importantly, apart from being involved in myeloid cell development, PU.1 has been shown to control the cell‐type specific expression of LPS‐responsive genes by constitutively binding to enhancer elements of inflammation‐associated genes and thereby increasing their accessibility to inducible transcription factors such as NFκB or AP‐1 78. Hence, low PU.1 expression may—apart from regulating CD16 expression—promote the hyporesponsiveness of LPMO toward LPS by impairing transactivation of LPS inducible genes.

In addition to its effect on receptors mediating innate immune responses, n‐butyrate downregulates gene expression of xCT, the light chain of the cystine‐ glutamate transporter, on PBMO. XCT is required for the uptake of cystine by myeloid cells, which subsequently release cysteine following reduction of the disulphide bond 12, 79, 80. The supply of cysteine is a prerequisite for T cells to synthesize the tripeptide glutathione, which is required by T cells to mount a proliferative response 80, 81. Down‐regulation of xCT expression may therefore provide an explanation for the low capacity of n‐butyrate treated human monocytes to promote antigen receptor/CD3‐ or alloantigen‐driven T cell proliferation as observed previously 82

In conclusion, the commensal microbiota—through the release of the short chain fatty acid n‐butyrate—appears to contribute to intestinal homeostasis by inducing a hyporesponsive state in intestinal macrophages through downregulation of CD11b, CD14, CD16 as well as the cystine‐glutamate transporter xCT. Regarding inhibition of CD16 gene expression, this study mechanistically suggests a sequence of events in which a critical transcription factor for this gene, PU.1, is itself downregulated at the transcript level by n‐butyrate likely through inhibition of HDACs.

## Conflicts of Interest

The authors declare no conflicts of interest.

## Supporting information

Additional supporting information may be found in the online version of this article at the publisher's web‐site.

**Figure S1**. Viability of PBMO cultured in the absence or presence of n‐butyrate. PBMO were cultured in the absence or presence of different concentrations of n‐butyrate (1 mM, 0.5 mM) for 24 h. Apoptosis of PBMO was detected by annexin V staining and subsequent flow cytometric analysis. Results are presented as % annexin V^+^ PBMO. Shown are the means ± SEM as well as individual data points of 5 independent experiments

**Figure S2**. n‐butyrate downregulates surface expression of innate response receptors in isolated primary human PBMO. A: PBMO were enriched by plastic adherence and subsequently cultured in the absence or presence of different concentrations of n‐butyrate (1 mM, 0.5 mM) for 24 h. Surface expression levels of CD11b, CD14, CD16 as well as HLA‐DR were analyzed on Annexin V^−^ PBMO using flow cytometry. Results are presented as % MFI relative to untreated control (medium). Shown are the results of two independent experiments. B: PBMO were purified using MACS® technology (negative selection) and subsequently cultured in the absence or presence of different concentrations of n‐butyrate (1 mM, 0.5 mM) for 24 h. Surface expression levels of CD11b, CD14, CD16 as well as HLA‐DR were analyzed on Annexin V‐ PBMO using flow cytometry. Results are presented as % MFI relative to untreated control (medium). Shown are the results of two independent experiments
