# Supplementary figures and images for: Human monocytes downregulate innate response receptors following exposure to the microbial metabolite n‐butyrate
Source: Immun Inflamm Dis. 2017 Jul 6;5(4):480–92. doi: 10.1002/iid3.184 (PMC5691313; doi:10.1002/iid3.184)

# Figure S1

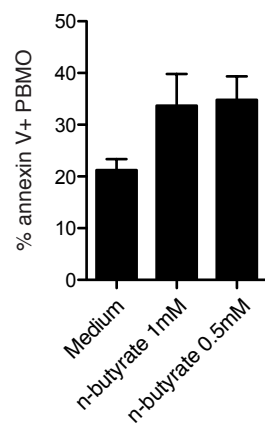

Supplement: Supplementary file 1 — Figure S1. Viability of PBMO cultured in the absence or presence of n‐butyrate. PBMO were cultured in the absence or presence of different concentrations of n‐butyrate (1 mM, 0.5 mM) for 24 h. Apoptosis of PBMO was detected by annexin V staining and subsequent flow cytometric analysis. Results are presented as % annexin V+ PBMO. Shown are the means ± SEM as well as individual data points of 5 independent experiments [file IID3-5-480-s001.pdf]

Figure S2A

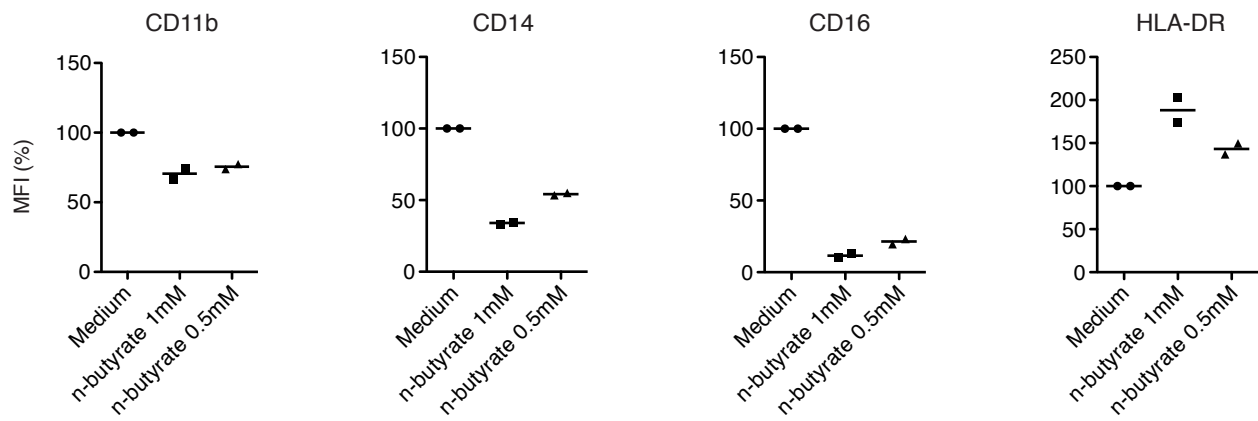

Figure S2B

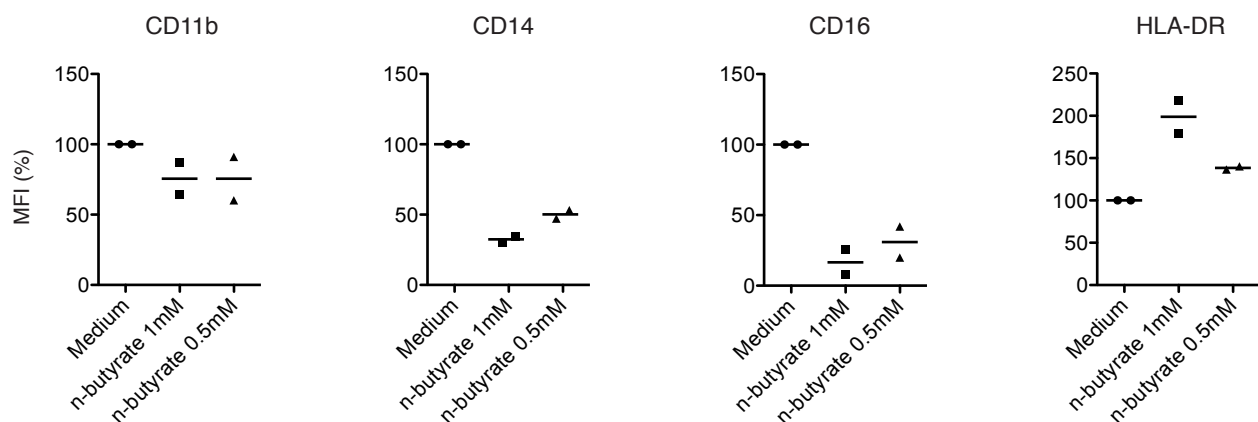

Supplement: Supplementary file 2 — Figure S2. n‐butyrate downregulates surface expression of innate response receptors in isolated primary human PBMO. A: PBMO were enriched by plastic adherence and subsequently cultured in the absence or presence of different concentrations of n‐butyrate (1 mM, 0.5 mM) for 24 h. Surface expression levels of CD11b, CD14, CD16 as well as HLA‐DR were analyzed on Annexin V− PBMO using flow cytometry. Results are presented as % MFI relative to untreated control (medium). Shown are the results of two independent experiments. B: PBMO were purified using MACS® technology (negative selection) and subsequently cultured in the absence or presence of different concentrations of n‐butyrate (1 mM, 0.5 mM) for 24 h. Surface expression levels of CD11b, CD14, CD16 as well as HLA‐DR were analyzed on Annexin V‐ PBMO using flow cytometry. Results are presented as % MFI relative to untreated control (medium). Shown are the results of two independent experiments [file IID3-5-480-s002.pdf]
